# The association between body fat distribution and bone mineral density: evidence from the US population

**DOI:** 10.1186/s12902-022-01087-3

**Published:** 2022-07-04

**Authors:** Ming Ma, Xiaolong Liu, Gengxin Jia, Bin Geng, Yayi Xia

**Affiliations:** 1 Department of Orthopaedics, Gansu Key Laboratory of Orthopaedics, Lanzhou University Second Hospital, No. 82 Cuiyingmen, Chengguan District, Lanzhou City, 730000 Gansu Province China; 2 Second Clinical Medical School, Lanzhou University, No. 82 Cuiyingmen, Chengguan District, Lanzhou City, 730000 Gansu Province China; 3 Orthopaedic Clinical Medical Research Center, No. 82 Cuiyingmen, Chengguan District, Lanzhou City, 730000 Gansu Province China; 4 Technology Center for Intelligent Orthopedic Industry, No. 82 Cuiyingmen, Chengguan District, Lanzhou City, 730000 Gansu Province China

**Keywords:** Android fat, Gynoid fat, Fat distribution, NHANES, BMD

## Abstract

**Objective:**

To investigate the association between different body fat distribution and different sites of BMD in male and female populations.

**Methods:**

Use the National Health and Nutrition Examination Survey (NHANES) datasets to select participants. The weighted linear regression model investigated the difference in body fat and Bone Mineral Density (BMD) in different gender. Multivariate adjusted smoothing curve-fitting and multiple linear regression models were used to explore whether an association existed between body fat distribution and BMD. Last, a subgroup analysis was performed according to age and gender group.

**Results:**

Overall, 2881 participants were included in this study. Compared to males, female participants had lower BMD (*P* < 0.05) and higher Gynoid fat mass (*P* < 0.00001), while there was no difference between Android fat mass (*P* = 0.91). Android fat mass was positively associated with Total femur BMD (Males, β = 0.044, 95% CI = 0.037, 0.051, *P* < 0.00001; Females, β = 0.044, 95% CI = 0.039, 0.049, *P* < 0.00001), Femoral neck BMD (Males, β = 0.034, 95% CI = 0.027, 0.041, *P* < 0.00001; Females, β = 0.032, 95% CI = 0.027, 0.037, *P* < 0.00001), and Total spine BMD (Males, β = 0.036, 95% CI = 0.029, 0.044, *P* < 0.00001; Females, β = 0.025, 95% CI = 0.019, 0.031, *P* < 0.00001). The Gynoid fat mass, subgroup analysis of age and ethnicity reached similar results.

**Conclusion:**

Body fat in different regions was positively associated with BMD in different sites, and this association persisted in subgroup analyses across age and race in different gender.

**Supplementary Information:**

The online version contains supplementary material available at 10.1186/s12902-022-01087-3.

## Introduction

Obesity was one of the serious health concerns affecting the health of the global population [1], especially in the US [2]. It had been shown that the adverse effects of obesity might be related to fat distribution [3]. Android obesity (also known as abdominal obesity, apple-shaped obesity) was associated with increased cardiovascular risk [4], mortality [5], or hypertension [6]. However, other studies suggested that Gynoid obesity (also known as pear-shaped obesity) may be related to a reduced cardiovascular disease risk [7] and metabolic disease [8]. So, what was the effect of fat distribution on BMD without considering body lean weight? This topic remained insufficiently researched.

Most previous studies used Body Mass Index (BMI) to assess obesity and explore the association between BMI and BMD [9, 10] and concluded a positive association. Nevertheless, BMI was widely used because it was easy to calculate, but it did not distinguish between fat, muscle, and fat distribution in different body sites. Furthermore, the extant studies that had examined the association between body fat and BMD reached controversial conclusions. In studies of Chinese populations, some studies had concluded that body fat mass was positively associated with BMD in both men and women [11–13], while other studies had concluded that increased fat had a negative effect on BMD [14]. Differential findings across gender in studies of populations in Brazil [15], Japan [16], Australia [17], and elsewhere were also found.

Furthermore, some of the available studies suggested that there might be differences in fat distribution between males and females. Males tended to have “android” obesity, where fat was concentrated in the abdomen, while females tended to have “gynoid” obesity, with more fat in the hips and thighs [18, 19]. This gender difference in fat distribution might be related to congenital genetics [20] and acquired environment [21], but whether this potentially different fat distribution affected the BMD of the femur or lumbar spine in different gender had not been well studied.

Thus, this study aimed to investigate the association between body fat distribution (Android fat and Gynoid fat) and different sites of BMD (Femur and Lumbar spine) in different gender populations in the US. Moreover, we hypothesized that android fat mass might be associated with higher lumbar spine BMD, while gynoid fat mass associated with higher femur BMD in males and females.

## Methods

### Datasets sources

This cross-sectional research selected datasets from the NHANES project, a nationally representative project to evaluate the health and nutritional status in the US. Database data was open to all researchers worldwide and easily accessible from the Centers for Disease Control and Prevention (CDC) website. In this study, we used the NHANES 2013–2014 and NHANES 2017–2018, as these were the only two datasets that had data on both BMD and body fat mass. After the datasets were downloaded from the CDC website to personal devices, EmpowerStats software was applied to merge and analyze the data. The study was reviewed and approved by NCHS IRB/ERB, all participants signed informed consent forms, and all methods were performed following relevant guidelines and regulations.

### Participants eligible

**Fig. 1:**
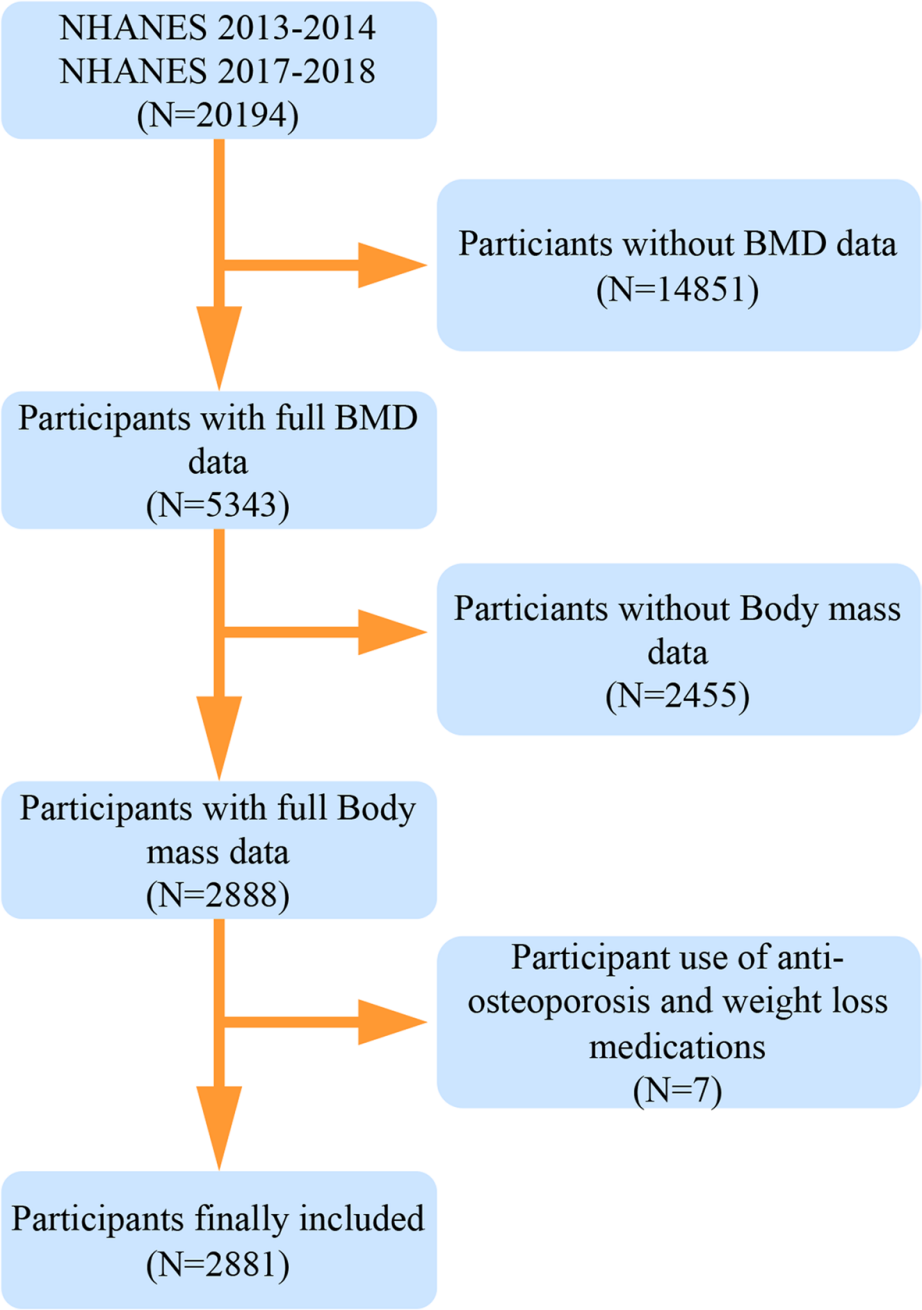
The participants selecting flow chart

Before the beginning of this study, the following people were not included: 1) Pregnant; 2) Received radiographic contrast agents in the past week; 3) Had body fat mass exceeding the device limits; 4) Had congenital malformations or degenerative diseases of the spine; 5) Had lumbar spinal surgery; 6) Had hip fractures or congenital malformations; 7) Had hip surgery; 8) Had implants in the spine, hip or body, or other problems affecting body measurements. From NHANES datasets, 20,194 participants were initially included in this study, 14,851 participants without femoral or lumbar spine BMD data, 2455 participants without body fat data, and 7 participants taking anti-osteoporosis or weight-loss pills were excluded. Eventually, a total of 2881 participants were included (Fig. 1).

### Exposure - body fat mass

The dual-energy X-ray absorptiometry (DXA) measured participants’ body fat mass [22]. The DXA model was Hologic QDR 4500A Fan Beam Bone Densitometer (Hologic, Inc., Bedford, Massachusetts), and the radiation dose of the equipment was less than 20uSv. The following methods were used for quality control: 1) monitoring of staff and machine operating conditions; 2) DXA scans followed standard radiological techniques, with expert review of all results to verify accuracy and consistency of results; 3) densitometers were calibrated daily through a rigorous body-mode scanning program, with longitudinal monitoring and cross-calibration of instruments at each site, using cumulative statistical methods (CUSUM) and Mobile Examination Center (MEC)-specific model data to identify breaks in densitometer calibration during the survey. The main measurements were Android/Gynoid fat mass, and the Hologic APEX software (Version 3.2) defined the Android/Gynoid regions [23]. The Android area was the area of the lower part of the trunk bounded by two lines: the horizontal cut line of the pelvis on its lower side and a line automatically placed above the pelvic line. Gynoid was defined by an upper line and a lower line, with the upper line being 1.5 times the height of the Android area below the pelvic line and the lower line being twice the height of the Android area. Finally, the Android/Gynoid ratio calculation was performed from the measured Android and Gynoid data.

### Outcome – BMD

Participants’ BMD was also measured by DXA equipment. The BMD measurement device information was the Hologic QDR-4500A sector beam densitometer (Hologic, Inc., Bedford, Massachusetts), and the rest of the radiation values and accuracy monitoring were consistent with the body fat measurement device. The femur and lumbar spine were scanned, including the Total femur, Femoral neck, and Total spine regions. Quality control of staff, scanning instruments, and scanning results were performed throughout the scanning process.

### Covariates

The following covariates were selected: demographics (age, race, education level, and poverty ratio), personal habits (physical activity, smoke, and alcohol use), comorbidities (osteoporosis, high blood pressure, and diabetes), and body measurements (Height, Weight, Body Mass Index). Demographic characteristics, personal habits, and comorbidity results were obtained from questionnaires, and body measurements were obtained from machine measurements.

### Statistical analysis

All study models were analyzed in gender subgroups to explore whether a gender difference existed between body fat distribution and BMD. Continuous variables in participants’ demographic information were expressed as Mean +/− standard deviations (SD), and *P*-values were calculated using a weighted linear regression model. Dichotomous variables were expressed as percentages, and weighted chi-square tests were used to calculate *P*-values.

Smoothing curve fitting models were used to assess whether there was an association between Android fat mass, Gynoid fat mass, and Android to Gynoid ratio and BMD. If the smoothing curve fitting were meaningful, the multiple regression analysis models were used to analyze the association between body fat mass and BMD, and the results were expressed in terms of *β*, 95% confidence intervals (CI), and *P*-values. Adjustments of covariates in the above models were based on the following criteria: 1) the addition or removal of the variable from the model had an effect of more than 10% on the coefficient value of body fat mass; 2) the covariate *P* < 0.1 in univariate model vs BMD (Supplementary File 1).

Finally, age and race analyses under different gender subgroups were performed with the same analytical models as above. All analyses were performed with R software (3.6.3 version) and EmpowerStats software (https://www.empowerstats.com). *P* < 0.05 was considered statistically significant.

## Results

### Characteristics of the selected participants

The basic characteristics of the participants were shown in Table 1. A total of 2881 participants (1245 males, 1636 females; mean age: 49 years) were included in this study. Among male participants, 33.9% had a daily physical activity, only 0.2% had osteoporosis, 43.3% had high blood pressure, and 21.9% had diabetes. While for female participants, 12.2% had osteoporosis, 48.5% had high blood pressure, and 15.7% had diabetes (All *P*-values < 0.05). For body examination data, female participants had higher BMI, Gynoid fat mass, and lower BMD when compared to the male participants (All *P*-values < 0.05).

**Table 1:** The characteristics of the participants selected

	Male (*N* = 1245)	Female (*N* = 1636)	***P*** value
**Demographic Data**			
Age (Years)	49.344 ± 5.717	49.016 ± 5.681	0.12498
Race (%)			
Mexican American	7.783	7.115	0.64412
Other Hispanic	3.908	5.041	
Non-Hispanic White	66.650	66.031	
Non-Hispanic Black	13.719	13.926	
Other Race	7.941	7.887	
Poverty ratio	3.149 ± 1.723	2.858 ± 1.697	0.00001
Education level (%)			
< ninth grade	3.789	3.302	< 0.00001
ninth - eleven grade	10.476	10.935	
High school	23.181	21.038	
Some college	25.322	38.829	
College graduate	37.232	25.897	
**Personal habits**			
Physical activity (%)			
Yes	33.920	27.527	0.00021
No	66.080	72.473	
Smoke (%)			
Yes	47.632	46.321	0.48372
No	52.368	53.679	
Alcohol use days per year	2.826 ± 2.134	2.197 ± 2.096	< 0.00001
**Comorbidities**			
Osteoporosis (%)			
Yes	0.210	12.223	< 0.00001
No	99.790	87.777	
High blood pressure (%)			
Yes	43.423	48.587	0.00579
No	56.577	51.413	
Diabetes (%)			
Yes	21.900	15.749	0.00003
No	78.100	84.251	
**Body examination data**			
Height (cm)	176.766 ± 7.022	162.282 ± 6.906	< 0.00001
Weight (kg)	92.645 ± 20.987	80.028 ± 21.643	< 0.00001
BMI (kg/m^2^)	29.548 ± 6.049	30.285 ± 7.657	0.00513
Android fat mass (kg)	2.843 ± 1.364	2.849 ± 1.466	0.91001
Gynoid fat mass (kg)	4.204 ± 1.607	5.580 ± 2.029	< 0.00001
Android to Gynoid ratio	1.145 ± 0.185	0.927 ± 0.177	< 0.00001
Total femur BMD (g/cm^2^)	1.008 ± 0.146	0.949 ± 0.154	< 0.00001
Femoral neck BMD (g/cm^2^)	0.826 ± 0.140	0.795 ± 0.146	< 0.00001
Total spine BMD (g/cm^2^)	1.049 ± 0.162	1.036 ± 0.157	0.03418

Mean +/− SD for continuous variables. Weighted linear regression model calculated *P*-value

Percentage (%) for continuous variables. Weighted chi-square test model calculated *P*-value

### Multivariable associations

**Fig. 2:**
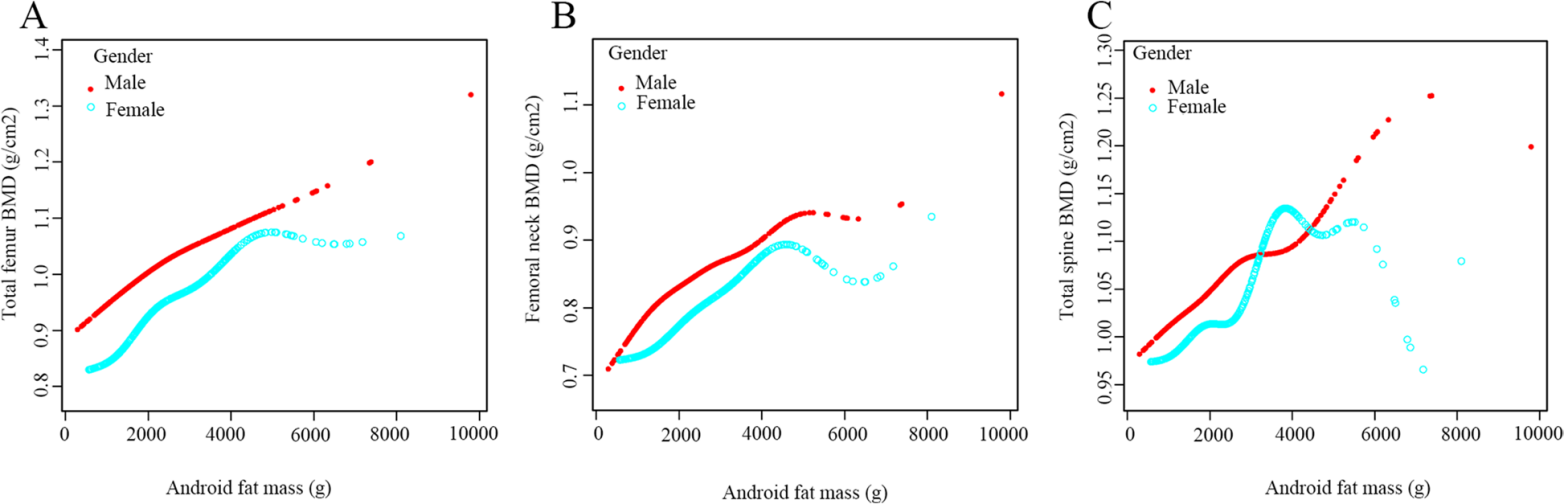
The association between Android fat mass and BMD. **A**. Total femur; (**B**). Femoral neck; (**C**). Total spine

**Fig. 3:**
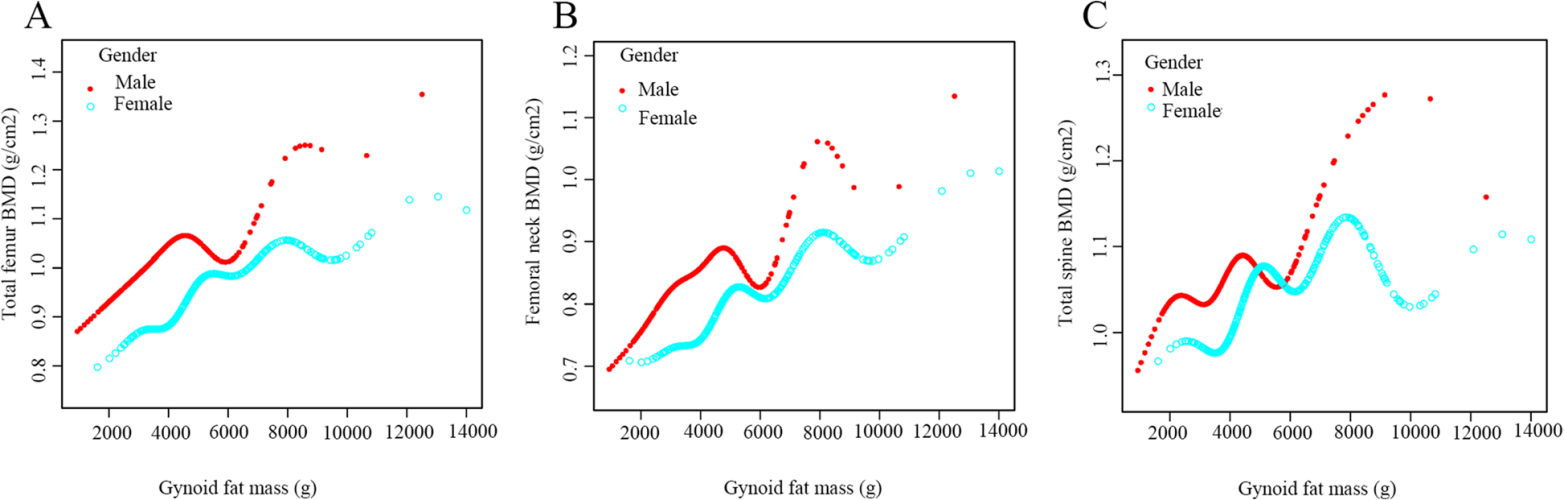
The association between Gynoid fat mass and BMD. **A**. Total femur; (**B**). Femoral neck; (**C**). Total spine

**Fig. 4:**
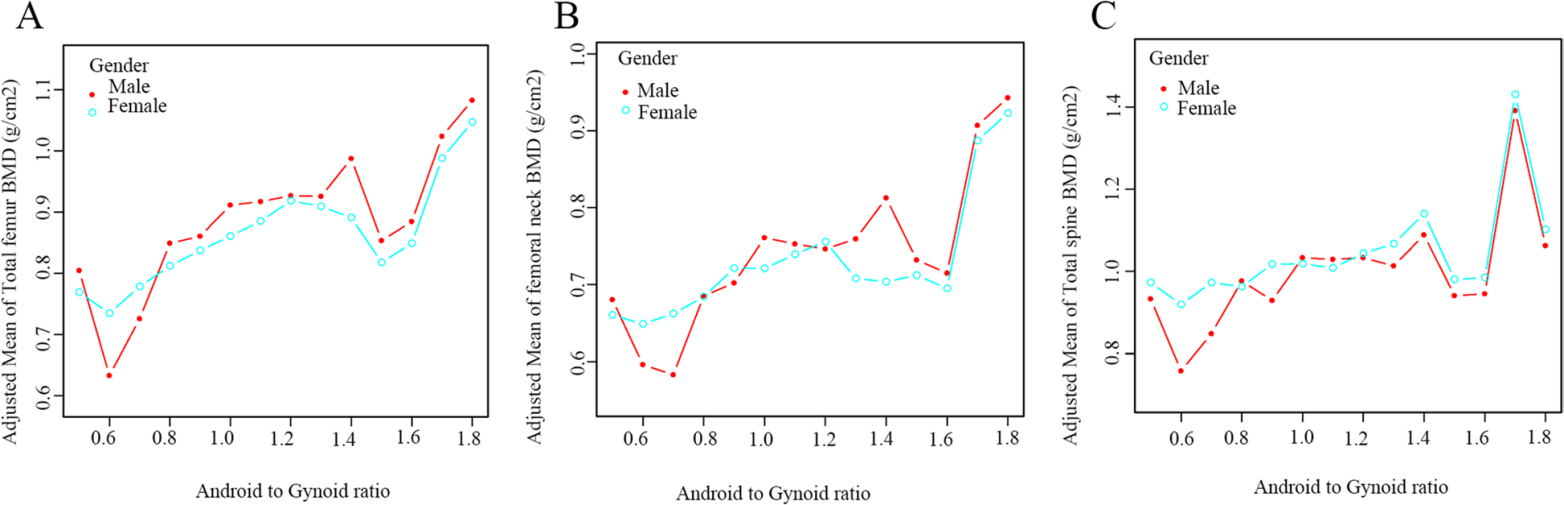
The association between Android to Gynoid ratio and BMD. **A**. Total femur; (**B**). Femoral neck; (**C**). Total spine

The multivariate-adjusted smoothed curve fitting models were used to investigate the association between Android fat mass, Gynoid fat mass and Android to Gynoid ratio and BMD in males and females. There was a linear positive association between Android fat mass and BMD in each region, regardless of male or female (Fig. 2). Similarly, there was also a linear positive association between Gynoid fat mass and individual regional BMD in different gender participants (Fig. 3). However, there was no apparent curvilinear association between the Android to Gynoid ratio and BMD in each region in males or females (Fig. 4).

Furthermore, multiple linear regression models were used to assess the specific β values and 95% CI between body fat mass and BMD in different gender (Table 2). Android fat mass was positively associated with Total femur BMD, Femoral neck BMD and Total spine BMD. Similarly, there was a similar positive association between Gynoid fat mass and BMD in both males and females (Results were shown in Table 2).

**Table 2:** The association between Android/Gynoid fat mass and BMD in different gender

	Model	Android fat mass (kg)		Gynoid fat mass (kg)	
		Male	Female	Male	Female
Total femur BMD (g/cm2)	Model I	0.048 (0.043, 0.053) < 0.00001	0.059 (0.054, 0.063) < 0.00001	0.045 (0.041, 0.050) < 0.00001	0.042 (0.039, 0.045) < 0.00001
	Model II	0.048 (0.042, 0.053) < 0.00001	0.050 (0.046, 0.054) < 0.00001	0.043 (0.038, 0.047) < 0.00001	0.035 (0.032, 0.038) < 0.00001
	Model III	0.044 (0.037, 0.051) < 0.00001	0.044 (0.039, 0.049) < 0.00001	0.039 (0.034, 0.045) < 0.00001	0.030 (0.026, 0.033) < 0.00001
Femoral neck BMD (g/cm2)	Model I	0.035 (0.030, 0.041) < 0.00001	0.044 (0.040, 0.048) < 0.00001	0.036 (0.032, 0.041) < 0.00001	0.038 (0.035, 0.040) < 0.00001
	Model II	0.035 (0.030, 0.040) < 0.00001	0.035 (0.031, 0.039) < 0.00001	0.034 (0.029, 0.038) < 0.00001	0.030 (0.027, 0.033) < 0.00001
	Model III	0.034 (0.027, 0.041) < 0.00001	0.032 (0.027, 0.037) < 0.00001	0.030 (0.025, 0.036) < 0.00001	0.028 (0.024, 0.031) < 0.00001
Total spine BMD (g/cm2)	Model I	0.047 (0.041, 0.053) < 0.00001	0.043 (0.038, 0.048) < 0.00001	0.044 (0.039, 0.049) < 0.00001	0.033 (0.030, 0.037) < 0.00001
	Model II	0.048 (0.042, 0.054) < 0.00001	0.035 (0.031, 0.040) < 0.00001	0.043 (0.037, 0.048) < 0.00001	0.026 (0.023, 0.030) < 0.00001
	Model III	0.036 (0.029, 0.044) < 0.00001	0.025 (0.019, 0.031) < 0.00001	0.032 (0.026, 0.039) < 0.00001	0.020 (0.016, 0.025) < 0.00001

All results were expressed as β (95% CI), *P*-value

Model I: No covariates were adjusted

Model II: Adjusted for Age and Race

Model III: Adjusted according to Supplementary File 1

### Subgroup analysis

In different age groups, Android fat mass (Males, Supplementary Table 1, Supplementary Fig. 1; Females, Supplementary Table 2, Supplementary Fig. 2) and Gynoid fat mass (Males, Supplementary Table 1, Supplementary Fig. 3; Females, Supplementary Table 2, Supplementary Fig. 4) were positively associated with BMD. In different race groups, Android fat mass (Males, Supplementary Table 3, Supplementary Fig. 5; Females, Supplementary Table 2, Supplementary Fig. 6) and Gynoid fat mass (Males, Supplementary Table 3, Supplementary Fig. 7; Females, Supplementary Table 1, Supplementary Fig. 8) were also positively associated with BMD.

## Discussion

In this US population-based cross-sectional research, we investigated the difference in body fat distribution in different gender and the association between body fat mass and BMD. There was a positive association between body fat distribution (Android and Gynoid) and BMD at each site (Femur and Lumbar spine) in both males and females. There was no difference in Android fat between participants by gender (*P* = 0.91), while the female participant group had higher Gynoid fat (*P* < 0.00001). Lastly, this association persisted when subgroup analyses for age and race were performed.

The main finding of this study was that body fat mass (Android or Gynoid) was positively associated with BMD, regardless of gender (Males or Females) or sites (Femur or Lumbar spine), which was inconsistent with our hypothesis or conventional perception. Gender differences were found in body fat distribution, consistent with the previous studies [24, 25]. In males, fat was more likely to be concentrated in the abdomen (Android fat), and in females, fat was more likely to be concentrated in the buttocks (Gynoid fat) [26]. Genome-wide association studies from the UK Biobank suggested that specific loci might determine fat distribution [27]. On the other hand, gene-environment-related effects were one of the possible mechanisms. Metabolomics [28], microbiomics [29], and the dietary lifestyle of individuals might all be involved.

The positive association was similar to the conclusions reached by numerous previous studies, for example, in Asian regions [11, 16, 30], and European regions [31, 32]. Also, some studies have concluded that there was no association or negative association between fat distribution and BMD [33–35]. Possible reasons for the inconsistent conclusions drawn from the above studies were as follows: 1) the sample size was too small, with most studies including only tens or hundreds of samples; 2) differences in age, gender, and ethnicity of the included participants; 3) differences in adjusted covariates when performing correlation analyses; and 4) other unknown reasons. Several possible explanations for the higher body fat mass associated with higher BMD. First, the more body fat there was, the greater the mechanical load on the bones. The mechanical load was very important for BMD maintenance [36, 37], and BMD would also decrease if one lost weight [38] or were in a weightless environment [39]. Second, hormones in high body fat individuals were important for protecting BMD. Estrogen was an early discovery of adipocyte-derived hormone, where androgens in adipocytes were transformed into estrogen by the action of aromatase [40, 41]. In addition, other hormones such as leptin [42] and insulin [43] were also involved in the adipose-bone mechanistic process. Finally, adipocytes and bone cells had a common origin from mesenchymal stem cells, and to some extent, adipogenesis and osteogenesis were dynamic processes involving multiple factors [44, 45].

The clinical significance of the present study was that, among other diseases, obesity could be considered a heterogeneous disease, where different body fat distribution might produce completely different or even opposite effects [46, 47]. However, for bone BMD, all were positively correlated and did not vary by the sites (femur or lumbar spine) or other differences (sex, age and race). Existing studies were not well explicit in exploring the association between fat distribution and BMD, and the lack of mechanistic studies made it difficult to explain this phenomenon. One possible reason was that, in the elderly, android fat and gynoid fat were interlinked and interconvertible [48]. Another possible explanation was that whether android fat or gynoid fat, they both had endocrine functions that produced estrogen, leptin, and others that had beneficial impacts on Bone [49]. In the future, more studies were needed to investigate the underlying reasons for the positive effect of body fat distribution on BMD.

In the end, the subgroup analysis led to the same conclusion. This indicated that the effect of body fat distribution on BMD was also not significantly related to age and race.

The strengths of this study were the following: 1) a representative large sample study; 2) the association of fat distribution (Android and Gynoid) on BMD at different sites (Femur and Lumbar spine) was explored in different gender populations; 3) adjusted for multiple covariates; 4) subgroup analysis was performed. In fact, the limitations of this study were as follows: 1) although the final number of participants included in this study was 2881, subsequent studies with larger samples were needed to continue validation; 2) this study was a cross-sectional and more future research were needed; 3) due to the limitations of the database itself, the menstrual status, participants’ hormone levels, whether the female participants were menopausal, and were not known, which might have unpredictable results on the female population impact; and 4) although many covariates were adjusted, there were still unknowable covariates. Therefore, to the best of our knowledge, the results of this study needed to be interpreted with caution.

## Conclusion

In this US population-based study, we found that Android/Gynoid fat mass was positively associated with femur/lumbar spine BMD. In addition, this positive correlation was also present in subgroups of age and race. However, the positive association between fat distribution and BMD was unrelated to sites (Femur or Lumbar spine) or gender (Males or Females).

## Supplementary Information

**Additional file 1.**

**Additional file 2.**

**Additional file 3.**

**Additional file 4.**

**Additional file 5.**

**Additional file 6.**

**Additional file 7.**

**Additional file 8.**

**Additional file 9.**

**Additional file 10.**

**Additional file 11.**

**Additional file 12.**

**Additional file 13.**

## Data Availability

The datasets used and analyzed during the current study are available from the corresponding author on reasonable request. For detailed information, see the NHANES website: https://www.cdc.gov/nchs/nhanes.

## Abbreviations

NHANES: National Health and Nutrition Examination Survey
BMD: Bone mineral density
BMI: Body mass index
DXA: Dual-energy X-ray
CI: Confidence Intervals
SD: Standard Deviations

## References

[1] Jaacks LM, Vandevijvere S, Pan A, McGowan CJ, Wallace C, Imamura F (2019). The obesity transition: stages of the global epidemic. Lancet Diabetes Endocrinol.

[2] Wang Y, Beydoun MA, Min J, Xue H, Kaminsky LA, Cheskin LJ (2020). Has the prevalence of overweight, obesity and central obesity levelled off in the United States? Trends, patterns, disparities, and future projections for the obesity epidemic. Int J Epidemiol.

[3] Ashwell M (1994). Obesity in men and women. Int J Obes Relat Metab Disord.

[4] Pischon T, Boeing H, Hoffmann K, Bergmann M, Schulze MB, Overvad K (2008). General and abdominal adiposity and risk of death in Europe. N Engl J Med.

[5] Zong G, Zhang Z, Yang Q, Wu H, Hu FB, Sun Q (2016). Total and regional adiposity measured by dual-energy X-ray absorptiometry and mortality in NHANES 1999-2006. Obesity (Silver Spring).

[6] Selvaraj S, Martinez EE, Aguilar FG, Kim KY, Peng J, Sha J, et al. Association of central adiposity with adverse cardiac mechanics: findings from the hypertension genetic epidemiology network study. Circ Cardiovasc Imaging. 2016;9. 10.1161/circimaging.115.004396. 10.1161/CIRCIMAGING.115.004396 PMC4911824 27307550

[7] Wiklund P, Toss F, Jansson JH, Eliasson M, Hallmans G, Nordström A (2010). Abdominal and gynoid adipose distribution and incident myocardial infarction in women and men. Int J Obes (Lond).

[8] Folsom AR, Kushi LH, Anderson KE, Mink PJ, Olson JE, Hong CP (2000). Associations of general and abdominal obesity with multiple health outcomes in older women: the Iowa Women's health study. Arch Intern Med.

[9] Ma M, Feng Z, Liu X, Jia G, Geng B, Xia Y (2021). The saturation effect of body mass index on bone mineral density for people over 50 years old: a cross-sectional study of the US population. Front Nutr.

[10] Padwal R, Leslie WD, Lix LM, Majumdar SR (2016). Relationship among body fat percentage, body mass index, and all-cause mortality: a cohort study. Ann Intern Med.

[11] Fan J, Jiang Y, Qiang J, Han B, Zhang Q (2022). Associations of fat mass and fat distribution with bone mineral density in non-obese postmenopausal Chinese women over 60 years old. Front Endocrinol (Lausanne).

[12] Fu X, Ma X, Lu H, He W, Wang Z, Zhu S (2011). Associations of fat mass and fat distribution with bone mineral density in pre- and postmenopausal Chinese women. Osteoporos Int.

[13] Lv S, Zhang A, Di W, Sheng Y, Cheng P, Qi H (2016). Assessment of fat distribution and bone quality with trabecular bone score (TBS) in healthy Chinese men. Sci Rep.

[14] Yu Z, Zhu Z, Tang T, Dai K, Qiu S (2009). Effect of body fat stores on total and regional bone mineral density in perimenopausal Chinese women. J Bone Miner Metab.

[15] Chain A, Crivelli M, Faerstein E, Bezerra FF (2017). Association between fat mass and bone mineral density among Brazilian women differs by menopausal status: the Pró-Saúde study. Nutrition.

[16] Douchi T, Yamamoto S, Oki T, Maruta K, Kuwahata R, Nagata Y (2000). Relationship between body fat distribution and bone mineral density in premenopausal Japanese women. Obstet Gynecol.

[17] Yang S, Center JR, Eisman JA, Nguyen TV (2015). Association between fat mass, lean mass, and bone loss: the Dubbo osteoporosis epidemiology study. Osteoporos Int.

[18] Vogel JA, Friedl KE (1992). Body fat assessment in women. Special considerations. Sports Medicine (Auckland, NZ).

[19] Wells JCK (2007). Sexual dimorphism of body composition. Best Practice & Research. Clinical. Endocrinol Metab.

[20] Zillikens MC, Yazdanpanah M, Pardo LM, Rivadeneira F, Aulchenko YS, Oostra BA (2008). Sex-specific genetic effects influence variation in body composition. Diabetologia.

[21] Lovejoy JC, Sainsbury A (2009). Sex differences in obesity and the regulation of energy homeostasis. Obes Rev.

[22] Lu Y, Mathur AK, Blunt BA, Gluer CC, Will AS, Fuerst TP (1996). Dual X-ray absorptiometry quality control: comparison of visual examination and process-control charts. J Bone Miner Res.

[23] Shepherd JA, Fan B, Lu Y, Wu XP, Wacker WK, Ergun DL (2012). A multinational study to develop universal standardization of whole-body bone density and composition using GE Healthcare lunar and Hologic DXA systems. J Bone Miner Res.

[24] Min KB, Min JY (2015). Android and gynoid fat percentages and serum lipid levels in United States adults. Clin Endocrinol (Oxf).

[25] Dos Santos MR, da Fonseca GWP, Sherveninas LP, de Souza FR, Battaglia Filho AC, Novaes CE (2020). Android to gynoid fat ratio and its association with functional capacity in male patients with heart failure. ESC. Heart Fail.

[26] Camilleri G, Kiani AK, Herbst KL, Kaftalli J, Bernini A, Dhuli K (2021). Genetics of fat deposition. Eur Rev Med Pharmacol Sci.

[27] Rask-Andersen M, Karlsson T, Ek WE, Johansson Å (2019). Genome-wide association study of body fat distribution identifies adiposity loci and sex-specific genetic effects. Nat Commun.

[28] Li X, L. Qi.Gene-environment interactions on body fat distribution. Int J Mol Sci. 2019;20. 10.3390/ijms20153690. 10.3390/ijms20153690 PMC6696304 31357654

[29] Min Y, Ma X, Sankaran K, Ru Y, Chen L, Baiocchi M (2019). Sex-specific association between gut microbiome and fat distribution. Nat Commun.

[30] Marwaha RK, Garg MK, Tandon N, Mehan N, Sastry A, Bhadra K (2013). Relationship of body fat and its distribution with bone mineral density in Indian population. J Clin Densitom.

[31] Gonnelli S, Caffarelli C, Tanzilli L, Alessi C, Tomai Pitinca MD, Rossi S (2013). The associations of body composition and fat distribution with bone mineral density in elderly Italian men and women. J Clin Densitom.

[32] Zillikens MC, Uitterlinden AG, van Leeuwen JP, Berends AL, Henneman P, van Dijk KW (2010). The role of body mass index, insulin, and adiponectin in the relation between fat distribution and bone mineral density. Calcif Tissue Int.

[33] Aedo S, Blümel JE, Carrillo-Larco RM, Vallejo MS, Aedo G, Gómez GG (2020). Association between high levels of gynoid fat and the increase of bone mineral density in women. Climacteric.

[34] Zhang W, Ma X, Xue P, Gao Y, Wu X, Zhao J (2014). Associations between fat distribution and volumetric bone mineral density in Chinese adults. Endocrine.

[35] Liu YH, Xu Y, Wen YB, Guan K, Ling WH, He LP (2013). Association of weight-adjusted body fat and fat distribution with bone mineral density in middle-aged chinese adults: a cross-sectional study. PLoS One.

[36] Kazakia GJ, Tjong W, Nirody JA, Burghardt AJ, Carballido-Gamio J, Patsch JM (2014). The influence of disuse on bone microstructure and mechanics assessed by HR-pQCT. Bone.

[37] Lohman T, Going S, Pamenter R, Hall M, Boyden T, Houtkooper L (1995). Effects of resistance training on regional and total bone mineral density in premenopausal women: a randomized prospective study. J Bone Miner Res.

[38] Chen X, Zhang J, Zhou Z (2021). Changes in bone mineral density after weight loss due to metabolic surgery or lifestyle intervention in obese patients. Obes Surg.

[39] Coulombe JC, Senwar B, Ferguson VL (2020). Spaceflight-induced bone tissue changes that affect bone quality and increase fracture risk. Curr Osteoporos Rep.

[40] Kameda T, Mano H, Yuasa T, Mori Y, Miyazawa K, Shiokawa M (1997). Estrogen inhibits bone resorption by directly inducing apoptosis of the bone-resorbing osteoclasts. J Exp Med.

[41] McTernan PG, Anderson LA, Anwar AJ, Eggo MC, Crocker J, Barnett AH (2002). Glucocorticoid regulation of p450 aromatase activity in human adipose tissue: gender and site differences. J Clin Endocrinol Metab.

[42] Cornish J, Callon KE, Bava U, Lin C, Naot D, Hill BL (2002). Leptin directly regulates bone cell function in vitro and reduces bone fragility in vivo. J Endocrinol.

[43] Hickman J, McElduff A (1989). Insulin promotes growth of the cultured rat osteosarcoma cell line UMR-106-01: an osteoblast-like cell. Endocrinology.

[44] Chen Q, Shou P, Zheng C, Jiang M, Cao G, Yang Q (2016). Fate decision of mesenchymal stem cells: adipocytes or osteoblasts?. Cell Death Differ.

[45] Migliaccio S, Greco EA, Fornari R, Donini LM, Lenzi A (2011). Is obesity in women protective against osteoporosis?. Diabetes Metab Syndr Obes.

[46] Neeland IJ, Turer AT, Ayers CR, Berry JD, Rohatgi A, Das SR (2015). Body fat distribution and incident cardiovascular disease in obese adults. J Am Coll Cardiol.

[47] Britton KA, Massaro JM, Murabito JM, Kreger BE, Hoffmann U, Fox CS (2013). Body fat distribution, incident cardiovascular disease, cancer, and all-cause mortality. J Am Coll Cardiol.

[48] Schosserer M, Grillari J, Wolfrum C, Scheideler M (2018). Age-induced changes in white, Brite, and Brown adipose depots: a Mini-review. Gerontology.

[49] Sadie-Van Gijsen H, Crowther NJ, Hough FS, Ferris WF (2013). The interrelationship between bone and fat: from cellular see-saw to endocrine reciprocity. CMLS.

