# Supplementary material for: The association between body fat distribution and bone mineral density: evidence from the US population
Source: BMC Endocr Disord. 2022 Jul 4;22:170. doi: 10.1186/s12902-022-01087-3 (PMC9254427; doi:10.1186/s12902-022-01087-3)
Supplement: Supplementary file 1 — Additional file 1. [file 12902_2022_1087_MOESM1_ESM.docx]

**Selected covariates**

X: Android fat mass (g)

| Y | X | Selected covariates |
| --- | --- | --- |
| Total femur BMD | Android fat mass (g) | Gender Age Race Education level Poverty ratio Height Alcohol use Diabetes Osteoporosis Smoke |
| Femoral neck BMD | Android fat mass (g) | Gender Age Race Education level Poverty ratio Height Alcohol use Diabetes Osteoporosis Smoke |
| Total spine BMD | Android fat mass (g) | Gender Age Race Education level Poverty ratio Height Height Alcohol use Diabetes High blood pressure Osteoporosis Smoke |

Criteria: 1). add the covariate to the basic model or remove it from the full model, change X coeff. >10%; OR 2). the covariate P<0.1 in the univariate model vs. Y

X: Gynoid fat mass (g)

| Y | X | Selected covariates |
| --- | --- | --- |
| Total femur BMD | Gynoid fat mass (g) | Gender Age Race Education level Poverty ratio Height Alcohol use Diabetes Osteoporosis Smoke |
| Femoral neck BMD | Gynoid fat mass (g) | Gender Age Race Education level Poverty ratio Height Alcohol use Diabetes Osteoporosis Smoke |
| Total spine BMD | Gynoid fat mass (g) | Gender Age Race Education level Poverty ratio Height Alcohol use Diabetes High blood pressure Osteoporosis Smoke |

Criteria: 1). add the covariate to the basic model or remove it from the full model, change X coeff. >10%; OR 2). the covariate P<0.1 in the univariate model vs. Y

X: Android to Gynoid ratio

| Y | X | Selected covariates |
| --- | --- | --- |
| Total femur BMD | Android to Gynoid ratio | Gender Age Race Education level Poverty ratio Physical activity Height Alcohol use Diabetes High blood pressure Osteoporosis Smoke |
| Femoral neck BMD | Android to Gynoid ratio | Gender Age Race Education level Poverty ratio Physical activity Height Alcohol use Diabetes High blood pressure Osteoporosis Smoke |
| Total spine BMD | Android to Gynoid ratio | Gender Age Race Education level Poverty ratio Physical activity Height Alcohol use Diabetes High blood pressure Osteoporosis Smoke |

Criteria: 1). add the covariate to the basic model or remove it from the full model, change X coeff. >10%; OR 2). the covariate P<0.1 in the univariate model vs. Y
