# Supplementary material for: The association between body fat distribution and bone mineral density: evidence from the US population
Source: BMC Endocr Disord. 2022 Jul 4;22:170. doi: 10.1186/s12902-022-01087-3 (PMC9254427; doi:10.1186/s12902-022-01087-3)
Supplement: Supplementary file 2 — Additional file 2. [file 12902_2022_1087_MOESM2_ESM.docx]

**Supplementary Table 1**: The association between Android fat mass/Gynoid fat mass between BMD in age subgroup in male participants.

|  | Model | Android fat mass (kg) | | Gynoid fat mass (kg) | |
| --- | --- | --- | --- | --- | --- |
|  |  | Age <= 50 years | Age > 50 years | Age <= 50 years | Age > 50 years |
| Total femur BMD (g/cm2) | Model 1, β (95% CI),  P-value | 0.031 (0.025, 0.037) <0.00001 | 0.074 (0.065, 0.084) <0.00001 | 0.032 (0.026, 0.037) <0.00001 | 0.063 (0.055, 0.070) <0.00001 |
|  | Model 2, β (95% CI),  P-value | 0.035 (0.029, 0.041) <0.00001 | 0.072 (0.063, 0.081) <0.00001 | 0.035 (0.030, 0.040) <0.00001 | 0.058 (0.051, 0.065) <0.00001 |
|  | Model 3, β (95% CI),  P-value | 0.036 (0.030, 0.042) <0.00001 | 0.064 (0.055, 0.074) <0.00001 | 0.036 (0.030, 0.041) <0.00001 | 0.051 (0.044, 0.059) <0.00001 |
| Femoral neck BMD (g/cm2) | Model 1, β (95% CI),  P-value | 0.023 (0.017, 0.029) <0.00001 | 0.053 (0.043, 0.062) <0.00001 | 0.023 (0.018, 0.029) <0.00001 | 0.053 (0.046, 0.060) <0.00001 |
|  | Model 2, β (95% CI),  P-value | 0.027 (0.022, 0.033) <0.00001 | 0.050 (0.041, 0.059) <0.00001 | 0.027 (0.021, 0.032) <0.00001 | 0.046 (0.040, 0.053) <0.00001 |
|  | Model 3, β (95% CI),  P-value | 0.028 (0.022, 0.035) <0.00001 | 0.045 (0.035, 0.054) <0.00001 | 0.035 (0.029, 0.042) <0.00001 | 0.075 (0.067, 0.084) <0.00001 |
| Total spine BMD (g/cm2) | Model 1, β (95% CI),  P-value | 0.038 (0.031, 0.046) <0.00001 | 0.060 (0.049, 0.070) <0.00001 | 0.039 (0.033, 0.046) <0.00001 | 0.048 (0.039, 0.056) <0.00001 |
|  | Model 2, β (95% CI),  P-value | 0.042 (0.035, 0.049) <0.00001 | 0.056 (0.046, 0.067) <0.00001 | 0.042 (0.036, 0.048) <0.00001 | 0.041 (0.033, 0.049) <0.00001 |
|  | Model 3, β (95% CI),  P-value | 0.041 (0.034, 0.049) <0.00001 | 0.046 (0.035, 0.057) <0.00001 | 0.041 (0.035, 0.048) <0.00001 | 0.033 (0.024, 0.042) <0.00001 |

Model 1: No covariates was adjusted.

Model 2: Adjusted for Race.

Model 3: Adjusted according to **Supplementary File 1**.
