# Supplementary figures and images for: The association between body fat distribution and bone mineral density: evidence from the US population
Source: BMC Endocr Disord. 2022 Jul 4;22:170. doi: 10.1186/s12902-022-01087-3 (PMC9254427; doi:10.1186/s12902-022-01087-3)

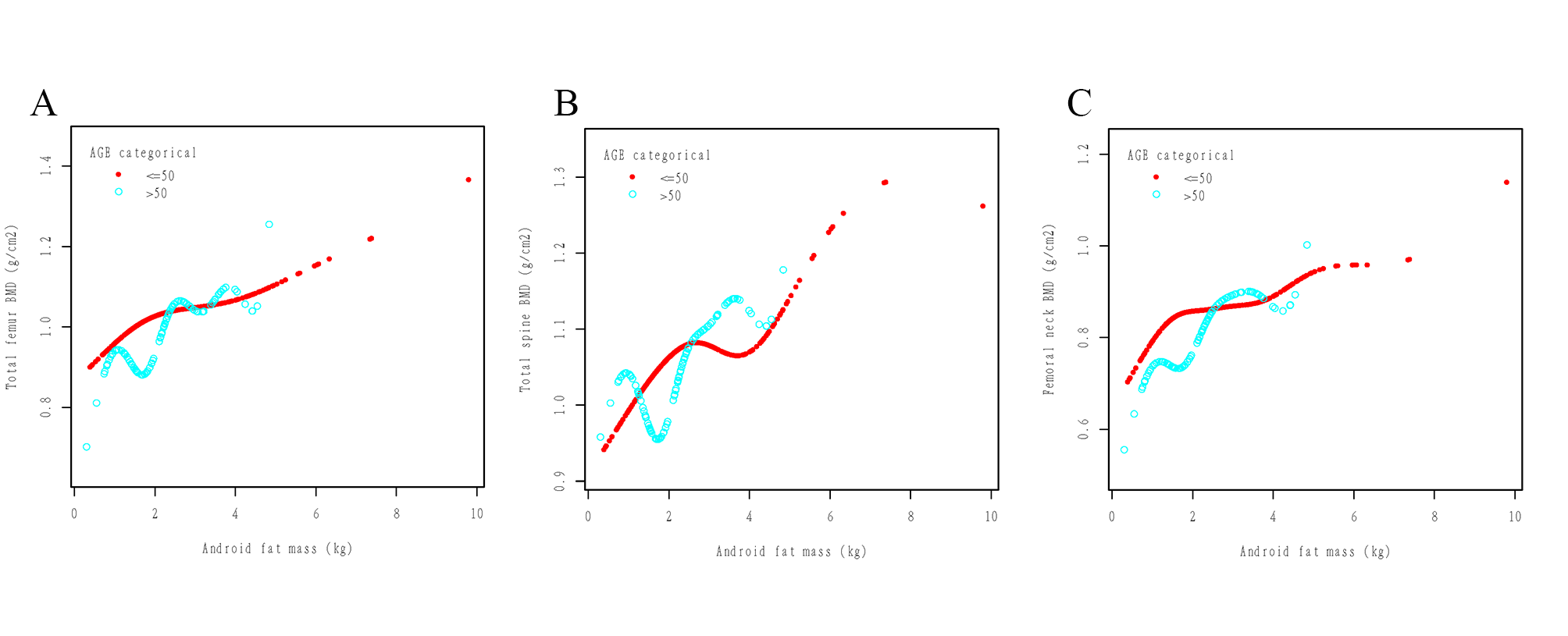

Supplement: Supplementary file 3 — Additional file 3. [file 12902_2022_1087_MOESM3_ESM.tif]

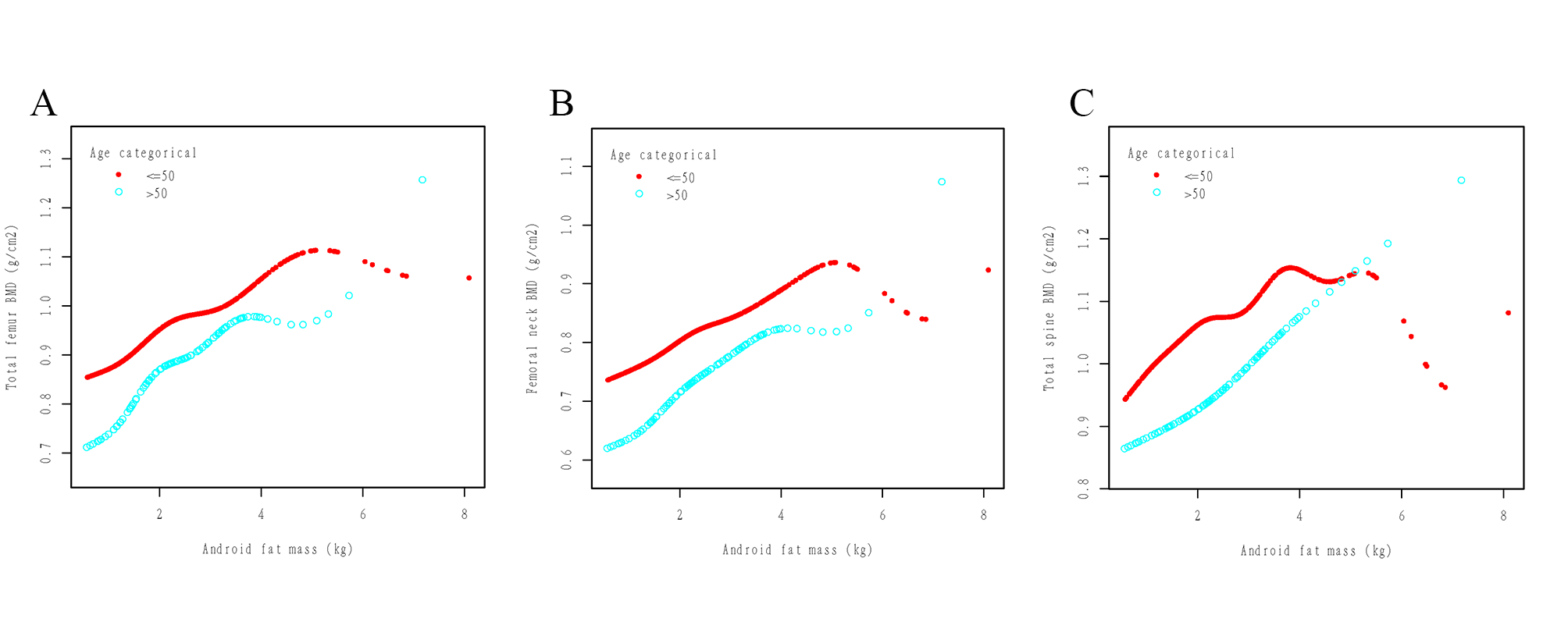

Supplement: Supplementary file 5 — Additional file 5. [file 12902_2022_1087_MOESM5_ESM.tif]

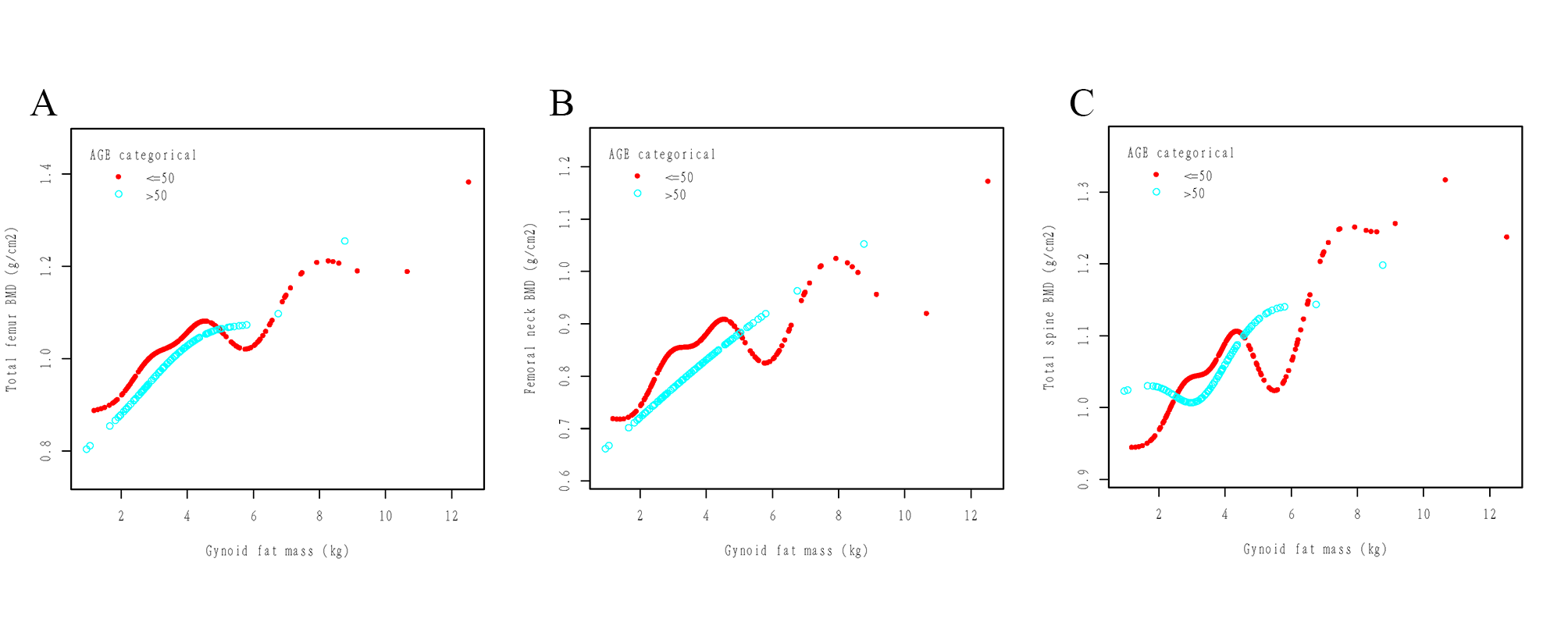

Supplement: Supplementary file 6 — Additional file 6. [file 12902_2022_1087_MOESM6_ESM.tif]

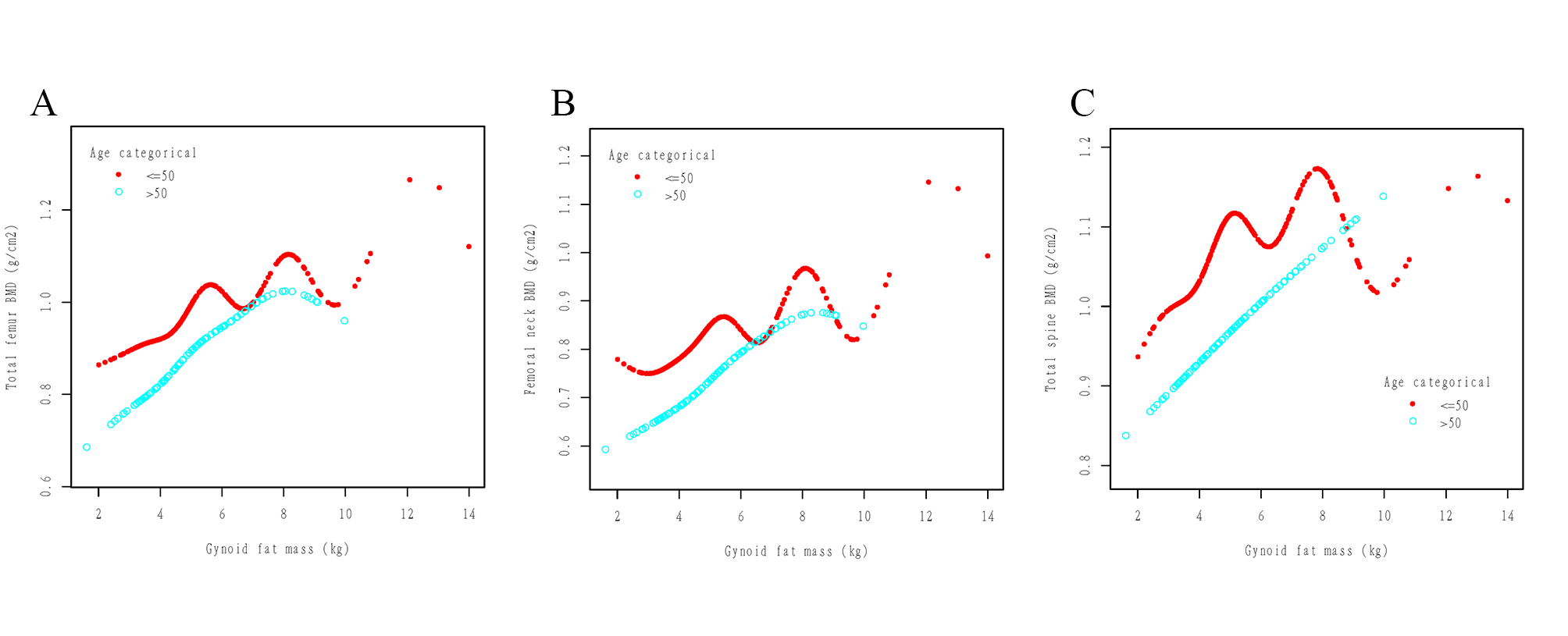

Supplement: Supplementary file 7 — Additional file 7. [file 12902_2022_1087_MOESM7_ESM.tif]

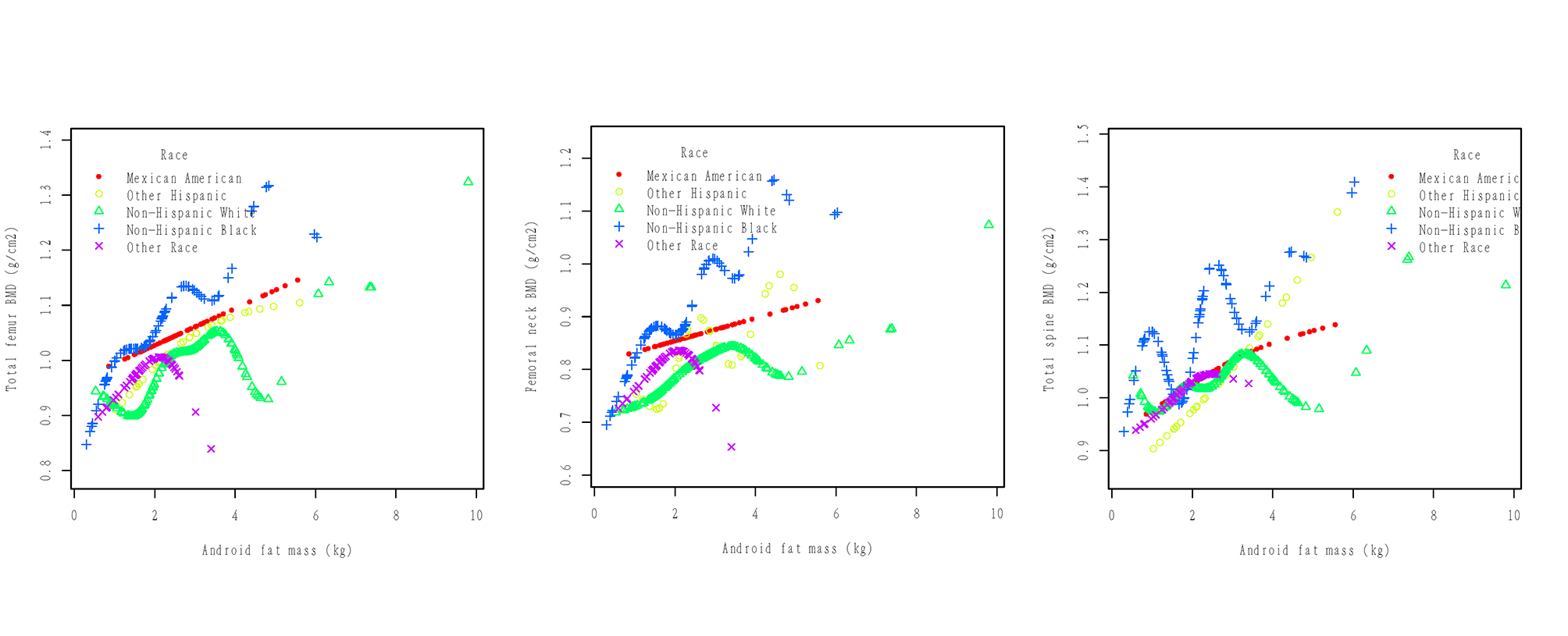

Supplement: Supplementary file 9 — Additional file 9. [file 12902_2022_1087_MOESM9_ESM.tif]

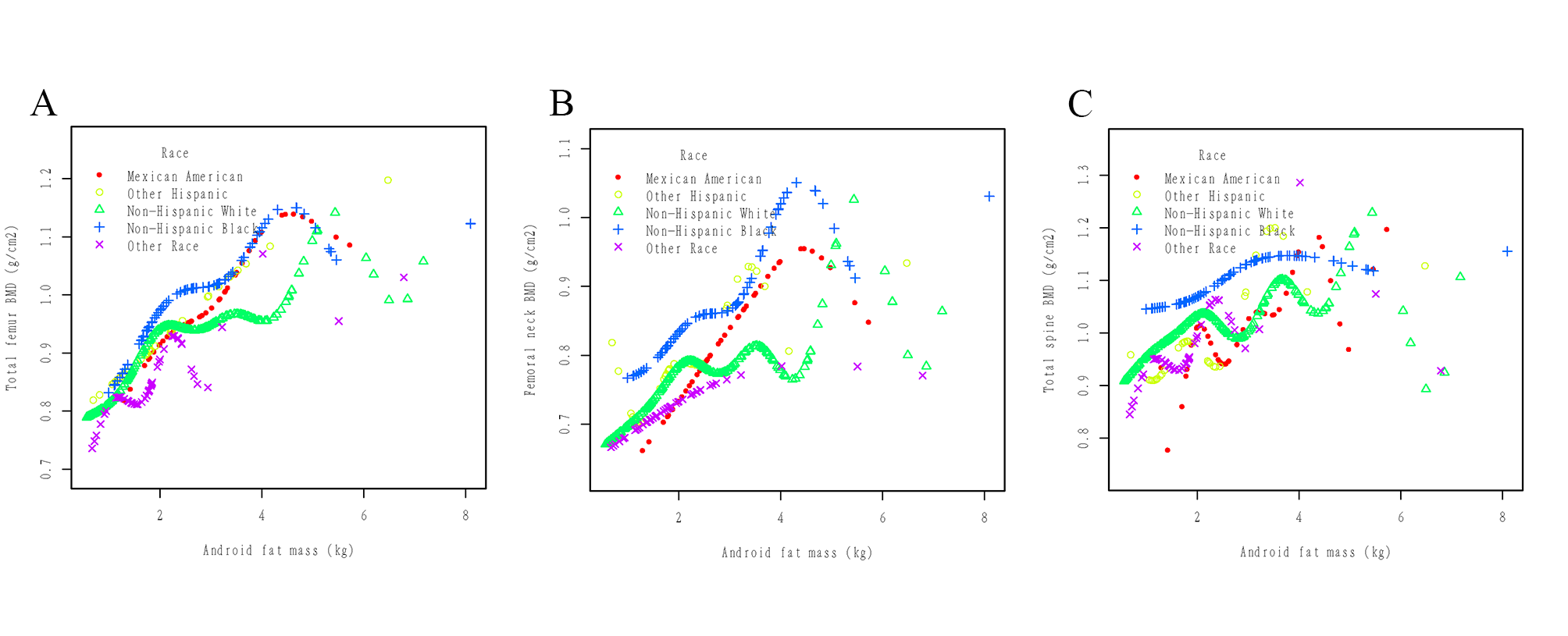

Supplement: Supplementary file 10 — Additional file 10. [file 12902_2022_1087_MOESM10_ESM.tif]

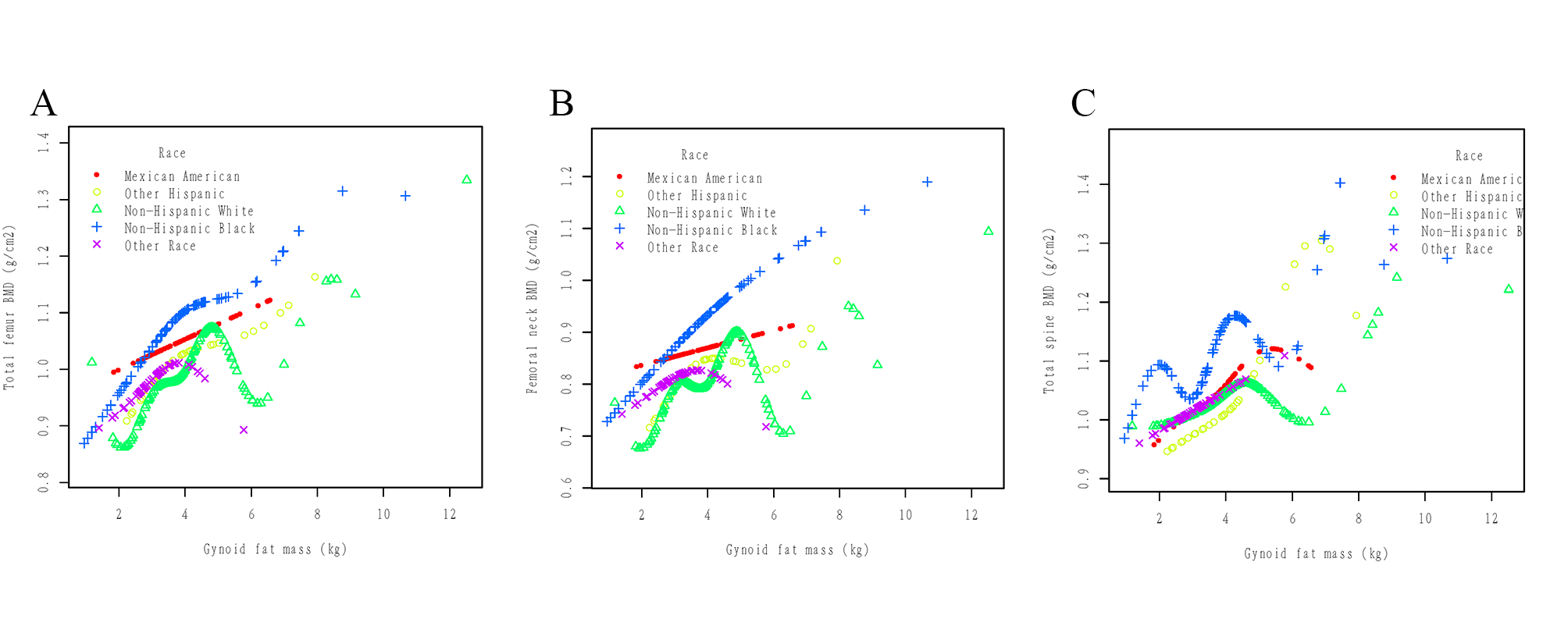

Supplement: Supplementary file 11 — Additional file 11. [file 12902_2022_1087_MOESM11_ESM.tif]

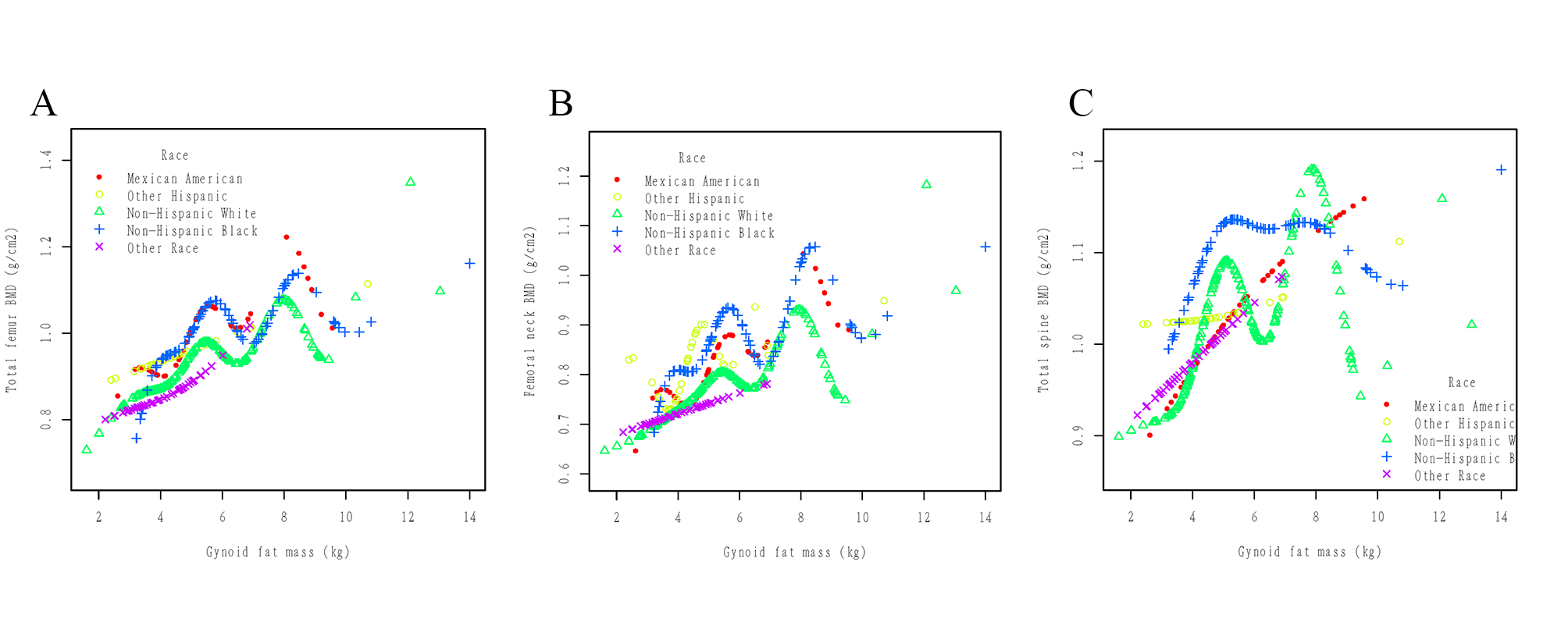

Supplement: Supplementary file 12 — Additional file 12. [file 12902_2022_1087_MOESM12_ESM.tif]
