# Supplementary material for: The association between body fat distribution and bone mineral density: evidence from the US population
Source: BMC Endocr Disord. 2022 Jul 4;22:170. doi: 10.1186/s12902-022-01087-3 (PMC9254427; doi:10.1186/s12902-022-01087-3)
Supplement: Supplementary file 4 — Additional file 4. [file 12902_2022_1087_MOESM4_ESM.docx]

**Supplementary Table 2**: The association between Android fat mass/Gynoid fat mass between BMD in age subgroup in female participants.

|  | Model | Android fat mass (kg) | | Gynoid fat mass (kg) | |
| --- | --- | --- | --- | --- | --- |
|  |  | Age <= 50 years | Age > 50 years | Age <= 50 years | Age > 50 years |
| Total femur BMD (g/cm2) | Model 1, β (95% CI),  P-value | 0.040 (0.035, 0.045) <0.00001 | 0.086 (0.079, 0.093) <0.00001 | 0.029 (0.025, 0.032) <0.00001 | 0.056 (0.052, 0.061) <0.00001 |
|  | Model 2, β (95% CI),  P-value | 0.040 (0.036, 0.045) <0.00001 | 0.082 (0.075, 0.089) <0.00001 | 0.029 (0.025, 0.033) <0.00001 | 0.055 (0.050, 0.060) <0.00001 |
|  | Model 3, β (95% CI),  P-value | 0.043 (0.038, 0.048) <0.00001 | 0.081 (0.074, 0.088) <0.00001 | 0.029 (0.025, 0.033) <0.00001 | 0.050 (0.045, 0.056) <0.00001 |
| Femoral neck BMD (g/cm2) | Model 1, β (95% CI),  P-value | 0.027 (0.021, 0.032) <0.00001 | 0.070 (0.063, 0.076) <0.00001 | 0.027 (0.023, 0.030) <0.00001 | 0.050 (0.046, 0.054) <0.00001 |
|  | Model 2, β (95% CI),  P-value | 0.027 (0.022, 0.032) <0.00001 | 0.066 (0.059, 0.073) <0.00001 | 0.027 (0.023, 0.031) <0.00001 | 0.049 (0.044, 0.053) <0.00001 |
|  | Model 3, β (95% CI),  P-value | 0.030 (0.025, 0.035) <0.00001 | 0.066 (0.060, 0.073) <0.00001 | 0.026 (0.022, 0.030) <0.00001 | 0.045 (0.041, 0.050) <0.00001 |
| Total spine BMD (g/cm2) | Model 1, β (95% CI),  P-value | 0.020 (0.014, 0.025) <0.00001 | 0.077 (0.070, 0.084) <0.00001 | 0.018 (0.014, 0.022) <0.00001 | 0.052 (0.047, 0.057) <0.00001 |
|  | Model 2, β (95% CI),  P-value | 0.019 (0.014, 0.025) <0.00001 | 0.072 (0.064, 0.079) <0.00001 | 0.017 (0.013, 0.022) <0.00001 | 0.048 (0.043, 0.053) <0.00001 |
|  | Model 3, β (95% CI),  P-value | 0.024 (0.018, 0.029) <0.00001 | 0.074 (0.067, 0.081) <0.00001 | 0.019 (0.015, 0.024) <0.00001 | 0.047 (0.041, 0.052) <0.00001 |

Model 1: No covariates was adjusted.

Model 2: Adjusted for Race.

Model 3: Adjusted according to **Supplementary File 1**.
