# Supplementary material for: The association between body fat distribution and bone mineral density: evidence from the US population
Source: BMC Endocr Disord. 2022 Jul 4;22:170. doi: 10.1186/s12902-022-01087-3 (PMC9254427; doi:10.1186/s12902-022-01087-3)
Supplement: Supplementary file 8 — Additional file 8. [file 12902_2022_1087_MOESM8_ESM.docx]

**Supplementary Table 3**: The association between Android fat mass/Gynoid fat mass between BMD in race subgroup in male participants.

|  | **Model** | **Race** | | | | |
| --- | --- | --- | --- | --- | --- | --- |
|  |  | Mexican American | Other Hispanic | Non-Hispanic White | Non-Hispanic Black | Other Race |
| **Android fat mass** (kg) | | | | | | |
| Total femur BMD (g/cm2) | Model 1, β (95% CI),  P-value | 0.028 (0.010, 0.046) 0.00304 | 0.048 (0.035, 0.061) <0.00001 | 0.046 (0.038, 0.053) <0.00001 | 0.068 (0.057, 0.080) <0.00001 | 0.079 (0.062, 0.095) <0.00001 |
|  | Model 2, β (95% CI),  P-value | 0.030 (0.013, 0.048) 0.00067 | 0.050 (0.037, 0.063) <0.00001 | 0.042 (0.034, 0.050) <0.00001 | 0.069 (0.058, 0.080) <0.00001 | 0.076 (0.060, 0.093) <0.00001 |
|  | Model 3, β (95% CI),  P-value | 0.039 (0.020, 0.058) 0.00008 | 0.034 (0.018, 0.050) 0.00009 | 0.039 (0.031, 0.046) <0.00001 | 0.070 (0.059, 0.081) <0.00001 | 0.074 (0.057, 0.091) <0.00001 |
| Femoral neck BMD (g/cm2) | Model 1, β (95% CI),  P-value | 0.009 (-0.009, 0.027) 0.33949 | 0.016 (0.004, 0.028) 0.01020 | 0.032 (0.025, 0.040) <0.00001 | 0.069 (0.058, 0.079) <0.00001 | 0.078 (0.062, 0.093) <0.00001 |
|  | Model 2, β (95% CI),  P-value | 0.013 (-0.004, 0.029) 0.13790 | 0.020 (0.008, 0.032) 0.00138 | 0.027 (0.020, 0.034) <0.00001 | 0.069 (0.059, 0.080) <0.00001 | 0.076 (0.060, 0.091) <0.00001 |
|  | Model 3, β (95% CI),  P-value | 0.027 (0.011, 0.044) 0.00178 | 0.018 (0.002, 0.035) 0.03324 | 0.025 (0.018, 0.033) <0.00001 | 0.070 (0.060, 0.081) <0.00001 | 0.072 (0.055, 0.088) <0.00001 |
| Total spine BMD (g/cm2) | Model 1, β (95% CI),  P-value | 0.048 (0.031, 0.065) <0.00001 | 0.082 (0.065, 0.098) <0.00001 | 0.039 (0.031, 0.047) <0.00001 | 0.061 (0.048, 0.074) <0.00001 | 0.139 (0.119, 0.158) <0.00001 |
|  | Model 2, β (95% CI),  P-value | 0.048 (0.031, 0.065) <0.00001 | 0.086 (0.070, 0.103) <0.00001 | 0.037 (0.028, 0.046) <0.00001 | 0.062 (0.049, 0.074) <0.00001 | 0.138 (0.119, 0.158) <0.00001 |
|  | Model 3, β (95% CI),  P-value | 0.039 (0.021, 0.057) 0.00004 | 0.079 (0.057, 0.101) <0.00001 | 0.033 (0.024, 0.042) <0.00001 | 0.061 (0.048, 0.074) <0.00001 | 0.126 (0.105, 0.148) <0.00001 |
| **Gynoid fat mass** (kg) | | | | | | |
| Total femur BMD (g/cm2) | Model 1, β (95% CI),  P-value | 0.021 (0.007, 0.034) 0.00273 | 0.043 (0.030, 0.057) <0.00001 | 0.044 (0.037, 0.051) <0.00001 | 0.049 (0.042, 0.057) <0.00001 | 0.067 (0.055, 0.080) <0.00001 |
|  | Model 2, β (95% CI),  P-value | 0.021 (0.008, 0.034) 0.00147 | 0.043 (0.030, 0.057) <0.00001 | 0.041 (0.034, 0.048) <0.00001 | 0.050 (0.043, 0.058) <0.00001 | 0.066 (0.053, 0.078) <0.00001 |
|  | Model 3, β (95% CI),  P-value | 0.024 (0.010, 0.038) 0.00093 | 0.026 (0.011, 0.042) 0.00169 | 0.038 (0.032, 0.045) <0.00001 | 0.052 (0.044, 0.060) <0.00001 | 0.064 (0.050, 0.078) <0.00001 |
| Femoral neck BMD (g/cm2) | Model 1, β (95% CI),  P-value | 0.009 (-0.005, 0.022) 0.21998 | 0.022 (0.011, 0.034) 0.00020 | 0.033 (0.027, 0.039) <0.00001 | 0.047 (0.040, 0.055) <0.00001 | 0.067 (0.055, 0.078) <0.00001 |
|  | Model 2, β (95% CI),  P-value | 0.009 (-0.003, 0.021) 0.15408 | 0.023 (0.012, 0.034) 0.00008 | 0.028 (0.022, 0.035) <0.00001 | 0.048 (0.041, 0.056) <0.00001 | 0.065 (0.054, 0.077) <0.00001 |
|  | Model 3, β (95% CI),  P-value | 0.015 (0.003, 0.027) 0.01894 | 0.026 (0.011, 0.040) 0.00115 | 0.027 (0.020, 0.033) <0.00001 | 0.050 (0.043, 0.058) <0.00001 | 0.063 (0.050, 0.076) <0.00001 |
| Total spine BMD (g/cm2) | Model 1, β (95% CI),  P-value | 0.034 (0.022, 0.047) <0.00001 | 0.076 (0.059, 0.093) <0.00001 | 0.037 (0.029, 0.044) <0.00001 | 0.041 (0.032, 0.050) <0.00001 | 0.116 (0.102, 0.130) <0.00001 |
|  | Model 2, β (95% CI),  P-value | 0.034 (0.022, 0.047) <0.00001 | 0.076 (0.059, 0.093) <0.00001 | 0.035 (0.027, 0.043) <0.00001 | 0.042 (0.033, 0.051) <0.00001 | 0.118 (0.103, 0.132) <0.00001 |
|  | Model 3, β (95% CI),  P-value | 0.028 (0.014, 0.041) 0.00007 | 0.062 (0.040, 0.085) <0.00001 | 0.032 (0.024, 0.040) <0.00001 | 0.043 (0.034, 0.052) <0.00001 | 0.110 (0.093, 0.127) <0.00001 |

Model 1: No covariates was adjusted.

Model 2: Adjusted for age.

Model 3: Adjusted according to **Supplementary File 1**.
