# Supplementary material for: The association between body fat distribution and bone mineral density: evidence from the US population
Source: BMC Endocr Disord. 2022 Jul 4;22:170. doi: 10.1186/s12902-022-01087-3 (PMC9254427; doi:10.1186/s12902-022-01087-3)
Supplement: Supplementary file 13 — Additional file 13. [file 12902_2022_1087_MOESM13_ESM.docx]

**Supplementary Table 4**: The association between Android fat mass/Gynoid fat mass between BMD in race subgroup in female participants.

|  | **Model** | **Race** | | | | |
| --- | --- | --- | --- | --- | --- | --- |
|  |  | Mexican American | Other Hispanic | Non-Hispanic White | Non-Hispanic Black | Other Race |
| **Android fat mass** (kg) | | | | | | |
| Total femur BMD (g/cm2) | Model 1, β (95% CI),  P-value | 0.086 (0.069, 0.103) <0.00001 | 0.065 (0.049, 0.082) <0.00001 | 0.064 (0.057, 0.070) <0.00001 | 0.032 (0.023, 0.040) <0.00001 | 0.043 (0.036, 0.051) <0.00001 |
|  | Model 2, β (95% CI),  P-value | 0.084 (0.068, 0.101) <0.00001 | 0.064 (0.049, 0.078) <0.00001 | 0.063 (0.057, 0.070) <0.00001 | 0.041 (0.033, 0.049) <0.00001 | 0.038 (0.030, 0.045) <0.00001 |
|  | Model 3, β (95% CI),  P-value | 0.070 (0.056, 0.084) <0.00001 | 0.050 (0.035, 0.066) <0.00001 | 0.062 (0.056, 0.069) <0.00001 | 0.043 (0.035, 0.052) <0.00001 | 0.048 (0.039, 0.057) <0.00001 |
| Femoral neck BMD (g/cm2) | Model 1, β (95% CI),  P-value | 0.073 (0.056, 0.091) <0.00001 | 0.069 (0.051, 0.087) <0.00001 | 0.048 (0.042, 0.054) <0.00001 | 0.025 (0.017, 0.033) <0.00001 | 0.013 (0.005, 0.021) 0.00176 |
|  | Model 2, β (95% CI),  P-value | 0.071 (0.054, 0.087) <0.00001 | 0.067 (0.052, 0.082) <0.00001 | 0.048 (0.041, 0.054) <0.00001 | 0.035 (0.027, 0.042) <0.00001 | 0.007 (-0.001, 0.015) 0.10401 |
|  | Model 3, β (95% CI),  P-value | 0.055 (0.041, 0.070) <0.00001 | 0.051 (0.036, 0.066) <0.00001 | 0.048 (0.041, 0.054) <0.00001 | 0.039 (0.030, 0.047) <0.00001 | 0.021 (0.011, 0.031) 0.00007 |
| Total spine BMD (g/cm2) | Model 1, β (95% CI),  P-value | 0.079 (0.063, 0.095) <0.00001 | 0.071 (0.049, 0.094) <0.00001 | 0.050 (0.043, 0.057) <0.00001 | 0.009 (0.001, 0.017) 0.02571 | 0.018 (0.006, 0.030) 0.00291 |
|  | Model 2, β (95% CI),  P-value | 0.076 (0.061, 0.091) <0.00001 | 0.068 (0.051, 0.086) <0.00001 | 0.049 (0.042, 0.056) <0.00001 | 0.017 (0.009, 0.024) 0.00003 | 0.012 (-0.000, 0.024) 0.05926 |
|  | Model 3, β (95% CI),  P-value | 0.060 (0.045, 0.075) <0.00001 | 0.054 (0.035, 0.072) <0.00001 | 0.049 (0.042, 0.057) <0.00001 | 0.019 (0.011, 0.027) <0.00001 | 0.017 (0.002, 0.032) 0.02531 |
| **Gynoid fat mass** (kg) | | | | | | |
| Total femur BMD (g/cm2) | Model 1, β (95% CI),  P-value | 0.050 (0.039, 0.061) <0.00001 | 0.036 (0.020, 0.051) <0.00001 | 0.047 (0.042, 0.051) <0.00001 | 0.018 (0.013, 0.024) <0.00001 | 0.047 (0.038, 0.055) <0.00001 |
|  | Model 2, β (95% CI),  P-value | 0.048 (0.037, 0.060) <0.00001 | 0.037 (0.024, 0.051) <0.00001 | 0.046 (0.041, 0.050) <0.00001 | 0.019 (0.014, 0.025) <0.00001 | 0.043 (0.035, 0.051) <0.00001 |
|  | Model 3, β (95% CI),  P-value | 0.036 (0.027, 0.046) <0.00001 | 0.032 (0.020, 0.044) <0.00001 | 0.042 (0.037, 0.047) <0.00001 | 0.020 (0.014, 0.026) <0.00001 | 0.051 (0.041, 0.061) <0.00001 |
| Femoral neck BMD (g/cm2) | Model 1, β (95% CI),  P-value | 0.049 (0.038, 0.060) <0.00001 | 0.034 (0.018, 0.051) 0.00007 | 0.041 (0.037, 0.045) <0.00001 | 0.019 (0.013, 0.024) <0.00001 | 0.018 (0.009, 0.027) 0.00018 |
|  | Model 2, β (95% CI),  P-value | 0.045 (0.034, 0.056) <0.00001 | 0.037 (0.022, 0.051) <0.00001 | 0.040 (0.036, 0.045) <0.00001 | 0.019 (0.014, 0.025) <0.00001 | 0.014 (0.006, 0.023) 0.00131 |
|  | Model 3, β (95% CI),  P-value | 0.031 (0.022, 0.041) <0.00001 | 0.033 (0.022, 0.045) <0.00001 | 0.037 (0.033, 0.042) <0.00001 | 0.022 (0.016, 0.027) <0.00001 | 0.025 (0.014, 0.036) <0.00001 |
| Total spine BMD (g/cm2) | Model 1, β (95% CI),  P-value | 0.050 (0.040, 0.060) <0.00001 | 0.025 (0.005, 0.045) 0.01745 | 0.037 (0.032, 0.042) <0.00001 | 0.008 (0.002, 0.013) 0.00629 | 0.041 (0.028, 0.054) <0.00001 |
|  | Model 2, β (95% CI),  P-value | 0.044 (0.034, 0.054) <0.00001 | 0.028 (0.012, 0.044) 0.00102 | 0.036 (0.031, 0.041) <0.00001 | 0.008 (0.003, 0.013) 0.00139 | 0.038 (0.025, 0.050) <0.00001 |
|  | Model 3, β (95% CI),  P-value | 0.031 (0.021, 0.041) <0.00001 | 0.023 (0.008, 0.038) 0.00316 | 0.033 (0.028, 0.038) <0.00001 | 0.010 (0.005, 0.016) 0.00015 | 0.039 (0.024, 0.054) <0.00001 |

Model 1: No covariates was adjusted.

Model 2: Adjusted for age.

Model 3: Adjusted according to **Supplementary File 1**.
